# Gene X Environment Interactions in Schizophrenia and Bipolar Disorder: Evidence from Neuroimaging

**DOI:** 10.3389/fpsyt.2013.00136

**Published:** 2013-10-14

**Authors:** Pierre Alexis Geoffroy, Bruno Etain, Josselin Houenou

**Affiliations:** ^1^U955, INSERM, Psychiatrie génétique, Créteil, France; ^2^AP-HP, Hôpital H. Mondor – A. Chenevier, Pôle de Psychiatrie, Créteil, France; ^3^Pôle de Psychiatrie, CHRU de Lille, Université Lille Nord de France, Lille, France; ^4^Fondation FondaMental, Créteil, France; ^5^Neurospin, CEA Saclay, Gif-Sur-Yvette, France

**Keywords:** schizophrenia, bipolar disorder, neuroimaging (MRI), gene, environment, GxE interaction

## Abstract

**Introduction:** Schizophrenia (SZ) and Bipolar disorder (BD) are considered as severe multifactorial diseases, stemming from genetic and environmental influences. Growing evidence supports gene x environment (GxE) interactions in these disorders and neuroimaging studies can help us to understand how those factors mechanistically interact. No reviews synthesized the existing data of neuroimaging studies in these issues.

**Methods:** We conduct a systematic review on the neuroimaging studies exploring GxE interactions relative to SZ or BD in PubMed.

**Results:** First results of the influence of genetic and environmental risks on brain structures came from monozygotic twin pairs concordant and discordant for SZ or BD. Few structural magnetic resonance imaging (sMRI) studies have explored the GxE interactions. No other imaging methods were found. Two main GxE interactions on brain volumes have arisen. First, an interaction between genetic liability to SZ and obstetric complications on gray matter, cerebrospinal fluid, and hippocampal volumes. Second, cannabis use and genetic liability interaction effects on cortical thickness and white matter volumes.

**Conclusion:** Combining GxE interactions and neuroimaging domains is a promising approach. Genetic risk and environmental exposures such as cannabis or obstetrical complications seem to interact leading to specific neuroimaging cerebral alterations in SZ. They are suggestive of GxE interactions that confer phenotypic abnormalities in SZ and possibly BD. We need further, larger neuroimaging studies of GxE interactions for which we may propose a framework focusing on GxE interactions data already known to have a clinical effect such as infections, early stress, urbanicity, and substance abuse.

## Introduction

Schizophrenia (SZ) and Bipolar disorder (BD) are considered as severe multifactorial diseases, stemming from genetic and environmental influences (1). SZ and BD are characterized by abnormalities of thought, behavior, cognition, and mood with overlap and similarities in the presentation and clinical course in many cases (2). Diagnostic instability in both disorders, frequency of schizoaffective cases, familial co-aggregation, and efficacy of new antipsychotics in both disorders challenged the traditional Kraepelinian binary classification of SZ and BD (2). Moreover, these observations of commonalities of SZ and BD are emphasized by growing and recent evidences from developmental, genetic, cognitive, neuroimaging, and environmental risk studies (3, 4). With regard to family studies, relatives of patients with SZ have an increased risk for both disorders with a higher risk for SZ (10%) than for BD (8%) and relatives of patients with BD have also an increased risk for both disorders with a higher risk for BD (10%) than for SZ (3.5%) (5). The largest available familial study observed an heritability for SZ and BD about 60% for both (with thus 40% for environmental effects) with partly shared genetic and environmental causes (1). Shared genetic effects for SZ that were in common with BD accounted for 52% of the genetic variance in SZ and for 69% in BD (1). Molecular studies demonstrated shared genetic etiology for SZ and BD with high genetic correlation (about 0.7) between both disorders (6). Moreover, prenatal maternal nutritional deficiency, maternal infection during pregnancy, season of birth, urbanicity, and obstetrical complications are environmental risk factors that have long been demonstrated and associated in both SZ and BD (7–15). These commonalities between SZ and BD can be also observed at the brain structure level. Indeed, the recent structural meta-analysis of controlled magnetic resonance imaging (sMRI) studies of De Peri et al. shows significant overall effect sizes for intracranial, whole brain, total gray and white matter (WM) volume reduction as well as for an increase of lateral ventricular volume at disease onset for both BD and SZ (16). Interestingly, some overlapping brain abnormalities may be already present at the onset of both diseases (16). However, both SZ and BD may present neurodevelopmental specificities as whole gray matter (GM) volume deficits and lateral ventricular enlargement that appear to be more prominent in first-episode SZ, whereas WM volume reduction seems more prominent in first-episode BD (16). These genetic and environmental influences with brain abnormalities might result in a cascade of events that manifest across a wide range of neurocognitive abnormalities in both SZ and BD that can be partly shared (17). However, worse performance exist in SZ, which can be recognized as a “generalized deficit,” compared to BD (17). This is particularly true for premorbid and current intelligence quotient and also perhaps attention, verbal memory, and executive functions (18). BD is rather characterized by generalized moderate level of neuropsychological impairment with deficits in some specific domains such as attention, executive function, and to a lesser extent verbal memory and spatial working memory (19).

As a simple binary classification of these two disorders seems an oversimplification of contemporary evidence, Insel and others have promoted the need to rethink investigative approaches and examine evidence of deficits or abnormalities in different research domains (e.g., cognitive, neuroimaging studies, environment risks) to build a dimensional picture of clinical presentations (20). This strategy helps clinicians identify similarities as well as differences between disorders and is in keeping with emerging research such as the family genetic studies of Lichtenstein and colleagues we previously presented (1).

To summarize, several lines of evidence support the neurodevelopmental hypothesis of SZ and BD where both diseases can be considered as pathophysiological processes starting early in life and resulting in pathological conditions during adulthood (21, 22). The implication of common genetic vulnerability and environmental risk factors, that may act early during development and even during fetal life, are well documented (23).

In this context, neuroimaging appears to be a relevant tool to identify putative intermediate phenotypes associated with neurodevelopmental abnormalities present in SZ and BD. Indeed, it is suggested that GM cortical reductions, cerebrospinal fluid (CSF), and hippocampal decrease volumes are associated with both SZ and BD patients and also observed among their relatives (24, 25). As we detailed previously, the common causative factors in SZ and BD, have been also observed with neuroimaging studies that showed substantial overlaps of brain abnormalities between both disorders (26). Further it is observed that brain volume is highly heritable in both disorders, but more consistently in SZ than in BD. Indeed, the Schizophrenia Twins and Relatives consortium recently observed that the heritability of most brain volumes were ranged between 52% (temporal cortical GM) to 76% (cerebrum) (27). Interestingly, it has been shown that the genetic influences and the disease-related deficits affect GM in partially non-overlapping areas of predominantly heteromodal association cortex, thus suggesting synergistic actions to produce the symptom severity and cognitive dysfunction of the disorder (28). First results of the influence of genetic and environmental risks on brain structures came from monozygotic twin pairs concordant and discordant for SZ or BD (29). These studies cannot specifically test the biological gene x environment (GxE) interaction effects, but twin studies might contribute to (1) validate the GxE interaction postulate, (2) quantify the relative effect of genetic and environmental factors on brain structures. Twin studies with monozygotic twin pairs concordant and discordant for SZ or BD, observe that total brain volume and most brain regional volumes are highly heritable (about 85%), whereas cerebral GM and lateral ventricles volumes showed more common environmental effects (about 65%) (27, 30–33).

Growing evidence supports GxE interactions in these disorders. But there are few studies about the mechanisms by which G and E interact in SZ and BD. To this purpose, neuroimaging studies can help us to understand how those factors mechanistically interact. To the best of our knowledge, this is the first review that synthesizes the existing data of neuroimaging studies of this issue.

## Methods

### Search strategy

We conducted in April 2013 a systematic review on the neuroimaging studies exploring GxE interactions associated to SZ or BD since 1990. The publications were obtained from the PubMed electronic database. The literature search was performed using the Mesh heading: (“schizophrenia” OR “bipolar disorder”) AND (“neuroimaging” OR “imagery” OR “MRI” OR “fMRI”) AND (“interaction” OR “GxE” OR “gene environment” OR “environment interaction”). The search found 202 results. Three supplementary original papers were identified through reviews.

### Study selection

We first removed duplicates. Then two authors (Pierre Alexis Geoffroy and Josselin Houenou) reviewed the title and/or abstracts of publications identified through databases to identify eligible studies. The two authors independently and then jointly selected studies for detailed extraction based on the full text. Studies were eligible if (1) they used neuroimaging exploration whatever was the imaging technique, (2) they were original studies without overlapping samples, and (3) reported a SZ or BD diagnosis confirmation.

**Figure 1:**
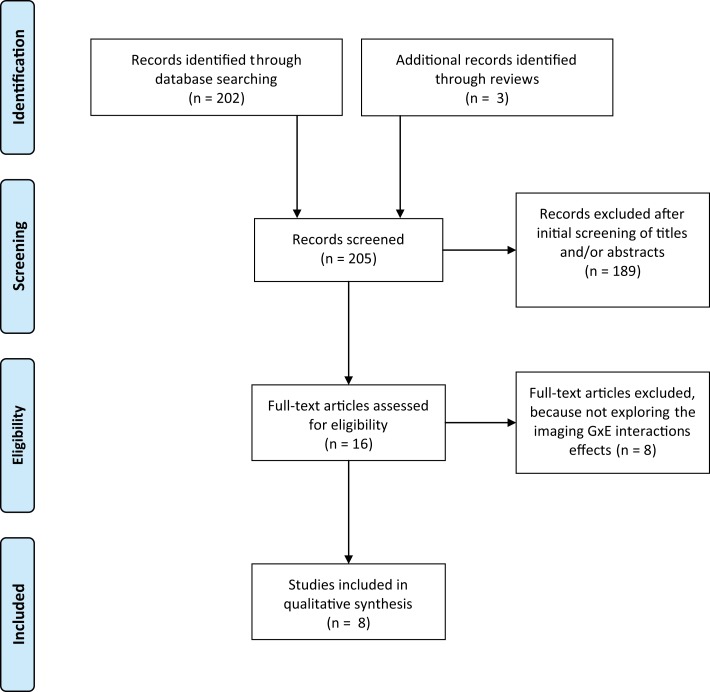
**Flow diagram of article selection process**. The literature search was performed from the PubMed electronic database and using the Mesh heading: (“schizophrenia” OR “Bipolar disorder”) AND (“neuroimaging” OR “imagery” OR “MRI” OR “fMRI”) AND (“interaction” OR “GxE” OR “Gene environment” OR “Environment interaction”).

Studies were excluded if (1) did not assessed GxE interactions in SZ or BD, (2) were review papers without new data, (3) were not written in English and/or published in peer-review journals. We followed the Preferred Reporting Items for Systematic Reviews and Meta-Analyses, referred as the PRISMA Statement (34). The Figure 1 shows the flow diagram of the review methods and the search strategy. At the end, eight studies were included in the qualitative analysis exploring specifically the imaging GxE interactions effects in SZ and/or BD (see Figure 1 for details).

## Results

Eight studies using structural MRI (sMRI) have explored the imaging GxE interactions in the physiopathology of SZ and schizoaffective disorder. No other imaging methods were found. Two main GxE interactions on brain volumes have arisen:

1. an interaction between genetic liability to SZ or schizoaffective disorder and obstetric complications (OCs).
2. an interaction between genetic liability to SZ or schizoaffective disorder and cannabis use.

### Obstetric complications and genetic liability interaction effects

Three classes of OCs have been studied: hypoxia-associated OCs, maternal infection during pregnancy, and maternal stress during pregnancy (35).

First results came from Cannon and colleagues in 1989 who used computed tomographic (CT). They measured ventricular, cortical, and cerebellar abnormalities to assess the effects of SZ genetic liability and perinatal complications (36). Later, the same research team found, with obstetric hospital records and brain magnetic resonance imaging scans (MRI), that the effect of birth complications on ventricular enlargement was greater among the offspring with two parents with SZ compared with those with one affected parent, who in turn had greater enlargement compared to those with healthy parents (37). They demonstrated that the interaction effects of the familial genetic risk for SZ and OC (as perinatal exposure to ether anesthesia) strongly predict the type and degree of brain abnormalities shown by adult subjects (37). Cannon and colleagues further clarified their findings comparing subjects with SZ or schizoaffective disorder, their siblings and healthy controls with the same records and MRI methods through two studies (38, 39). They replicated the environmental risk effect of fetal hypoxia that predicted reduced GM and increased CSF in the whole cortex (most strongly in the temporal lobe) in patients and their siblings but not among healthy controls at low risk for SZ (38). Haukvik and colleagues tested the interaction effects between severe fetal hypoxia and variation in four hypoxia-regulated SZ susceptibility genes (*BDNF*, *DTNBP1*, *GRM3*, and *NRG1*) on hippocampal volume in subjects with SZ and healthy controls (40). Of the 32 single nucleotides polymorphisms (SNPs) studied, an allele variation in *GRM3* rs13242038 was associated with effects of severe fetal hypoxia on hippocampal volume in this relatively small sample (54 patients with SZ and 53 controls) (40). This study is of interest because variation in hypoxia-regulated genes, in combination with severe OCs leading to hypoxia, is thought to increase the risk of SZ (41). Further, the effect of severe OCs on disease risk was shown to be modified by SNP variation in *BDNF*, *DTNBP1*, and *GRM3* (41). Thus interestingly this preliminary study shows that the effect of OCs on hippocampal volume could be modified by variation in hypoxia-regulated genes.

In sum, these studies converge toward the existence of an interaction between genetic liability to SZ or schizoaffective disorder and OCs (especially fetal hypoxia) on GM, CSF, and hippocampal volumes.

### Cannabis use and genetic liability interaction effects

Cannabis use during early adolescence is associated with a twofold increased risk for SZ in psychosis-free persons and with a poor prognosis for those with an established vulnerability to psychotic disorder (severe level of psychotic symptoms and increase need for care) (42). McGrath et al. recently precised, using sibling pair analysis in a cohort of young adults, that young adults who commenced cannabis use before 15 years were twice as likely to develop a non-affective psychosis (43). Further, longer duration since first cannabis use was associated with higher risk of psychosis-related outcomes by a “dose-response” relationship (43). The effects of exogenous cannabis and endo-cannabinoids are mostly mediated by cannabinoid receptor 1 (CNR1 or CB1R), which is widely expressed in the brain (44).

Habets and colleagues first examined the impact of cannabis use and developmental trauma associated with SZ on cortical thickness (45). These authors found for cannabis a significant interaction effect with stronger reductions of cortical thickness for both SZ groups and their healthy siblings than healthy controls, thus suggesting cannabis use and genetic liability interaction effects on the cerebral cortical thickness. An interaction effect between developmental trauma and cortical thickness was only observed in the SZ group but not with their healthy siblings and so did not demonstrate a GxE interaction as for cannabis use (45). Further, Ho and colleagues examined among subjects with SZ the interactions between *CNR1* genetic variants and heavy cannabis use on brain volumes and cognitive function (46). Significant *CNR1* genotype-by-cannabis use interaction effects were observed on WM volumes and neurocognitive impairment in patients with SZ suggesting that heavy cannabis use in the context of specific *CNR1* genotypes may contribute to confer WM abnormalities and cognitive impairment in SZ (46). The same team recently examined the effect of another cannabinoid-related gene, mitogen-activated protein kinase 14 (*MAPK14*): they assessed the *MAPK14*-*CNR1* gene–gene interactions in conferring brain volumes abnormalities among subjects with SZ with cannabis abuse (47). Authors observed significant main effects of the *MAPK14 CNR1* diplotype and diplotype × cannabis interaction on WM brain volumes. Further, the two genetic variants had additive contributions to WM volume deficits in SZ subjects with cannabis misuse but not in patients not using cannabis (47). Thus interestingly, these findings suggested a potential gene–gene interaction that influences brain volumes among subjects with SZ and cannabis use.

In sum, these studies showed that specific *CNR1* variants and potential gene–gene interactions interacted with cannabis use on WM brain volumes and cognitive functions among subjects with SZ.

## Discussion

**Figure 2:**
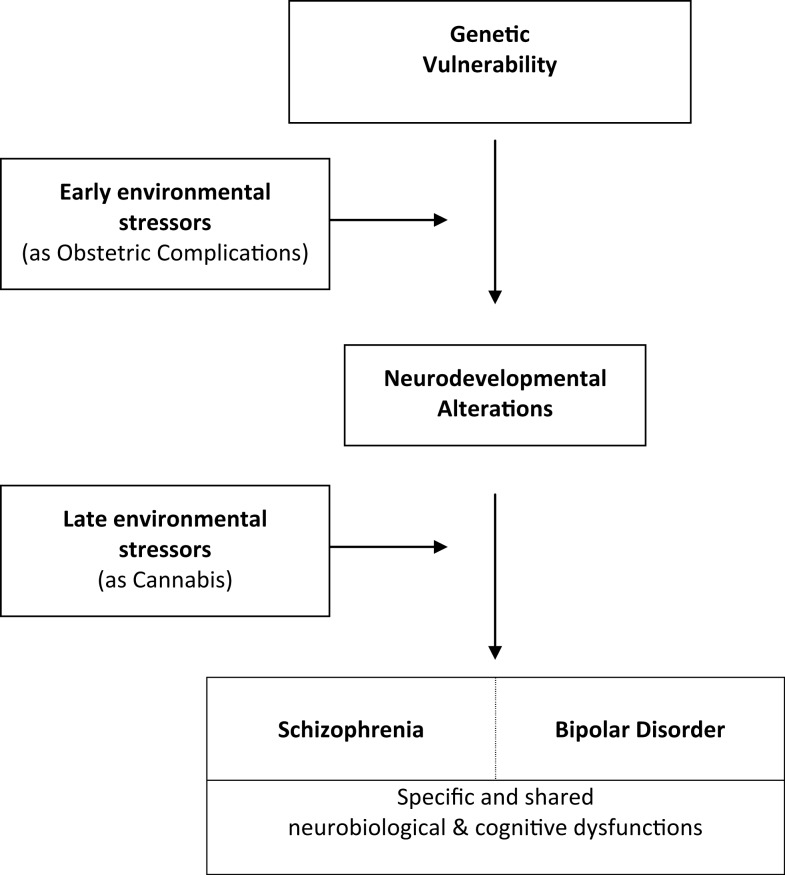
**Common developmental pathways leading to schizophrenia and bipolar disorder**.

With two distinct environmental events such as OCs and cannabis use, we observe significant early and late GxE interaction effects on WM volumes that lead to neurocognitive impairment conferring phenotypic abnormalities in SZ. No specific studies exist on BD, but the existence of an interaction between genetic liability to schizoaffective disorder and fetal hypoxia on GM, CSF, and hippocampal volumes is of interest and warrant further explorations in BD population. These findings further substantiate the hypothesis that brain abnormalities in SZ- and possibly BD- are at least in part neurodevelopmental in origin and arise from GxE interactions. We summarize these interactions in a putative common developmental pathway leading to both disorders (Figure 2). The key idea here is that genetically vulnerable populations may be more sensitive to environmental risk factors (48).

The field of GxE interactions on gray and WM is very promising but still poorly explored. Our review revealed that only very few environmental factors have been explored (mainly cannabis and obstetrical complications). Each of these factors has been studied by one or two groups at most, requiring replication studies. Other factors, such as migration, urbanicity, season of birth, early stress, or infections during pregnancy have not been explored despite being consistently associated with risk for SZ and BD (7–15). Further GxE interactions neuroimaging studies only used sMRI. However, other neuroimaging methods might be of interest as functional MRI (fMRI) that has demonstrated to be efficient in genetic and environmental explorations (49, 50). In addition, no study has assessed the GxE interactions with neuroimaging in BD. Nevertheless, these preliminary findings in SZ and schizoaffective disorder are paving the way to address more specifically this question in BD.

We need further neuroimaging studies for which we may propose a framework focusing on existing GxE interactions data. Such study is quite challenging, regarding the high number of environmental factors known to increase risk for SZ or BD (infections, season of birth, early stress, or abuse, urbanicity, substance abuse, migration). The tremendous number of genetic variants identified in BD and SZ is even higher, and still growing. It is therefore not conceivable to study all GxE interactions, as it would require very huge sample sizes, unrealistic for neuroimaging study.

We propose to focus on already clinically and epidemiologically known GxE interactions. They include interactions between genetic risk for SZ and BD and OCs (51, 52), urbanicity (53, 54), early stress (55–57), and cannabis. Other potential GxE interactions are more debated and are currently being assessed by large studies such as EU-GEI (58). Regarding genetic risk, we can assess differential effects of environment on MRI patterns between siblings of affected subjects and controls; or, within controls, between carriers of different allelic variants. Nevertheless, this second approach may be criticized as only very few genetic variants have consistently been shown to have GxE interaction effect. An intermediate solution may be the calculation of indirect measures of genetic risk such as a “genetic risk score” from GWAS data (6). However, none of the currently reported gene variants have come up as highly significant. Indeed, Candidate genes associated with BD and SZ display relatively low odds ratios (OR) and minor allele frequencies (MAF), and therefore it is unlikely that both disorders are determined by common variants with large effect sizes. Thus, future studies could benefit from investigations of polygenic risk scores, genetic pathways, and gene expressions rather than single variants.

Additionally, some studies have explored GxE interactions on cognition. A recent report on 234 adult healthy twins found a significant interaction between childhood maltreatment and *COMT* genotype on cognition (59). This was in accordance with a previous experimental study that reported a moderation of the effects of cannabis on cognition by the same *COMT* genotype (60). As cognitive performance is closely linked to neuroimaging features, such GxE interaction studies of cognition may bring us clues to potentially interesting GxE interactions to study with MRI.

The sample size required for such studies can also be decreased by cautious high-quality measurement of environmental risk factors, selection of frequent environmental risk factors, repeated measures, and selection of extreme exposure groups (58). Nevertheless, such GxE neuroimaging studies will probably require sample sizes that will need multi-site inclusion and imaging.

The identification of GxE interactions is reported to require at least a fourfold larger sample size than the identification of a main genetic effect of comparable magnitude (without including imagery, which increase this number) (61). Another solution can be to focus on recent findings on main genetics effects in SZ and/or BD (62).

In addition to sample size issues, future studies in order to increase their reliability will probably need to consider independent replication samples in their study design, which is basic standard by now in genetic studies. Several others methodological aspects will need to be addressed in the future as the imaging GxE corrections for multiple testing and issues such as low MAF and environmental exposure rates, the multitude of possible environmental influences on the genome (negative and positive influences), and the developmental stage of the individual since environmental experiences may differ in their impact (63).

Finally, we have to highlight that neuroimaging studies are relatively indirect measures of cellular activity and so cannot explain by themselves the underpinning mechanistic of these GxE interactions. But, multimodal human and animal studies can probably help address cellular and molecular mechanisms underlying brain activity (63). Indeed, molecular biology findings from animal studies can generate targets for human neuroimaging GxE research, which can focus on the genes of interest and neuroimaging probes of the relevant brain circuits. On the other hand, findings from human research can generate putative targets in animal models to study the underpinning molecular mechanisms of these neuroimaging GxE observations. Such translational researches would definitely help to better understand the whole picture of GxE interactions.

## Conclusion

Combining GxE interactions and neuroimaging domains is a promising approach. Genetic risk and environmental exposures, such as cannabis and obstetrical complications, seem to interact leading to specific neuroimaging cerebral alterations in SZ and possibly BD. We need replications and further large neuroimaging studies to explore the GxE interactions on brain anatomy and function.

## Conflict of Interest Statement

Pierre Alexis Geoffroy has received a price by Bayer for being Laureate of the medical university of Lille. Bruno Etain has received honoraria and financial compensation as independent symposium speakers from Sanofi-Aventis, Lundbeck, AstraZeneca, Eli Lilly, Bristol Myers Squibb, and Servier.
